# Glycogen Synthase Kinase 3β Modulates the Inflammatory Response Activated by Bacteria, Viruses, and Parasites

**DOI:** 10.3389/fimmu.2021.675751

**Published:** 2021-05-04

**Authors:** Ricarda Cortés-Vieyra, Octavio Silva-García, Anel Gómez-García, Sergio Gutiérrez-Castellanos, Cleto Álvarez-Aguilar, Víctor M. Baizabal-Aguirre

**Affiliations:** ^1^ División de Investigación Clínica, Centro de Investigación Biomédica de Michoacán, Instituto Mexicano del Seguro Social (IMSS), Morelia, Mexico; ^2^ Centro Multidisciplinario de Estudios en Biotecnología, Facultad de Medicina Veterinaria y Zootecnia, Universidad Michoacana de San Nicolás de Hidalgo, Morelia, Mexico; ^3^ Coordinación Auxiliar Médica de Investigación en Salud, IMSS Michoacán, Morelia, Mexico

**Keywords:** glycogen synthase kinase 3β, inflammation, inhibitors, bacteria, virus, parasites

## Abstract

Knowledge of glycogen synthase kinase 3β (GSK3β) activity and the molecules identified that regulate its function in infections caused by pathogenic microorganisms is crucial to understanding how the intensity of the inflammatory response can be controlled in the course of infections. In recent years many reports have described small molecular weight synthetic and natural compounds, proteins, and interference RNA with the potential to regulate the GSK3β activity and reduce the deleterious effects of the inflammatory response. Our goal in this review is to summarize the most recent advances on the role of GSK3β in the inflammatory response caused by bacteria, bacterial virulence factors (i.e. LPS and others), viruses, and parasites and how the regulation of its activity, mainly its inhibition by different type of molecules, modulates the inflammation.

## Introduction

Glycogen synthase kinase 3 (GSK3) is an evolutionarily conserved eukaryotic Ser/Thr kinase that regulates a broad range of substrates, which to date includes more than 100 proteins (1) with diverse function such as receptors, structural proteins, signaling molecules, and transcriptional factors, making GSK3 one of the most versatile kinases in the cell (2). This enzyme plays an important role in glycogen metabolism, cell cycle control, apoptosis, embryonic development, cell differentiation, cell motility, microtubule function, cell adhesion and inflammation (3, 4). The main isoforms of GSK3, GSK3α and GSK3β (5), are encoded by two different genes *gsk3α* and *gsk3β.* The isoforms share an identity of approximately 98% within their kinase domains and 100% similarity, being able to phosphorylate the same substrates (2). GSK3β is activated by phosphorylation at Tyr216 and it is inactivated by phosphorylation at Ser9. Given its involvement as repressors of several pathways such as apoptosis, insulin, phosphoinositide 3-kinase (PI3K), wingless and int-1 (Wnt)/β-catenin, hedgehog, and notch, this enzyme is involved in essentially every major process in the cell (3). Besides, GSK3β regulates many components of the innate and adaptive immune systems due to the modulation of a number of important transcription factors (6–8).

## GSK3β Transcriptionally Regulates Pro- and Anti-Inflammatory Responses

**Figure 1:**
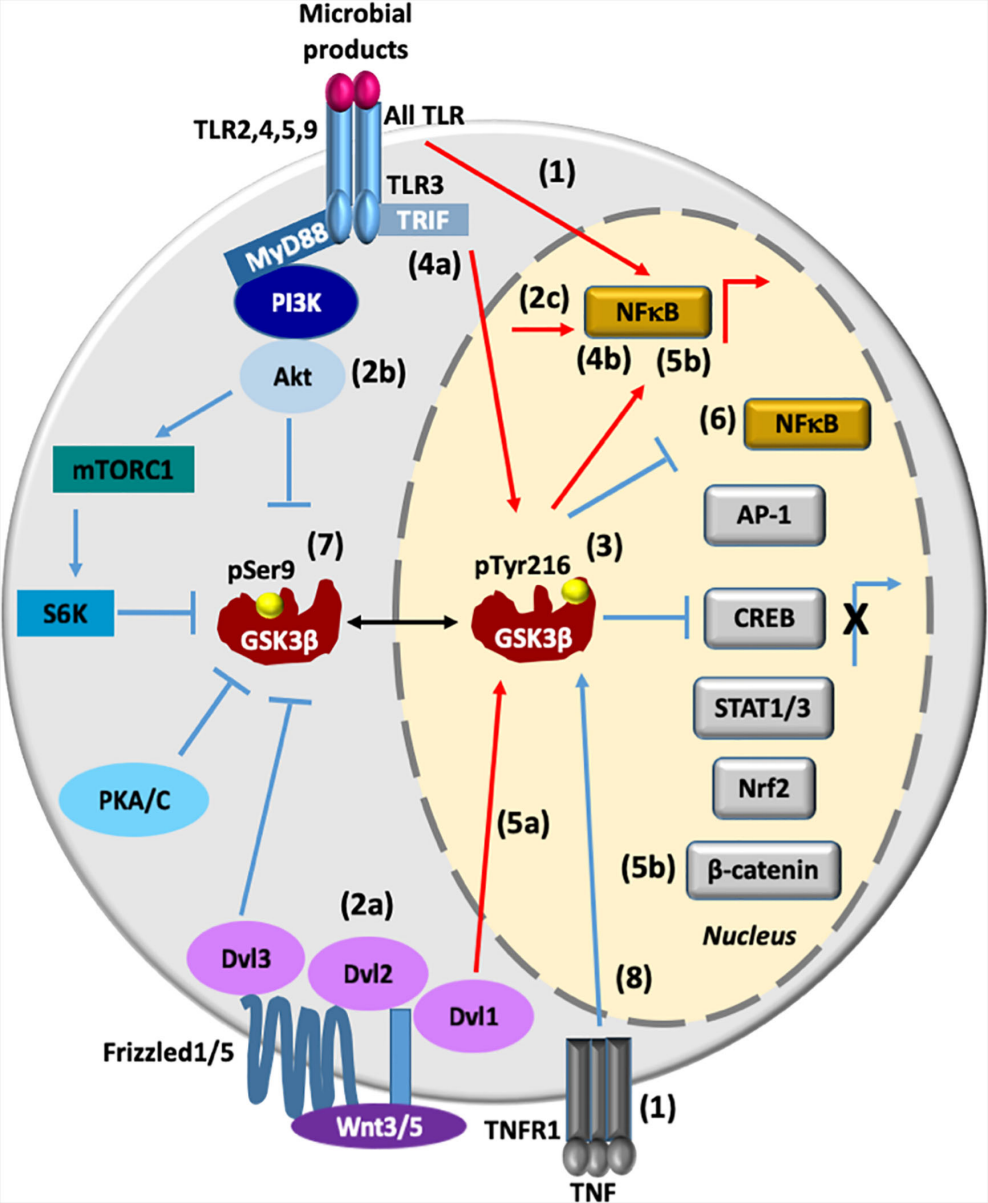
GSK3β transcriptionally regulates pro- and anti-inflammatory responses. (1). All TLR and TNFR1-associated intracellular signaling promote transcriptional activation of NF-κB. (2a-c) Wnt3/5/Dvl2/Akt signaling also promotes activation of NF-κB. (3) The active state of phosphorylated GSK3β at Tyr216(GSK3β pTyr216) promotes the activation of NF-κB and at the same time it promotes the inhibition of AP1, CREB, STAT1/3, Nrf2, and β-catenin, leading to proinflammatory cytokines production. (4a, b) During viral infection, TLR3/TRIF/GSK3β signaling pathway positively regulates both pro-inflammatory cytokines and IFN-β production through NF-κB and AP-1 activation and IRF3 activation, respectively. (5a, b) Activation of the canonical Wnt signaling pathway enhances activation of GSK3β, thus influencing β-catenin degradation and NF-κB activation (6). Overexpression of GSK3β inhibits NF-κB transcriptional activity. (7) Upon the activation of TLR (2, 4, 5, or 9)/MyD88/PI3K/Akt signaling, GSK3β is inhibited by phosphorylation at Ser9 (pSer9), leading to an anti-inflammatory response. GSK3β is also phosphoinhibited by S6K, PKA/C, and Dvl3 proteins. (8) TNF induces an increase in the nuclear expression of GSK3β and GSK3β promotes anti-inflammatory responses by mediating expression of the signaling inhibitors that terminate TLR4-induced NF-κB signaling, and by suppressing chromatin remodeling. Lines in red denote a proinflammatory response, while lines in blue denote an anti-inflammatory response. AP-1, activator protein 1; IRF3, interferon regulatory factor 3; CREB, cAMP-response element binding protein; NF-κB, nuclear factor kappa-light-chain-enhancer of activated B cells; Nrf2, nuclear factor erythroid 2-related factor 2; PI3K, phosphoinositide 3-kinase; PKA/C, protein kinase A/C; PKB, protein kinase B, also known as Akt; STAT1-3, signal transducers and activators of transcription 1-3; S6K, ribosomal protein 6 kinase; TLR2, 4, 5, 9, Toll-like receptor 2, 4, 5 and 9; TNFR, tumor necrosis factor receptor; Wnt5, wingless-related integration site member 5:: dishevelled segment polarity protein 1, 2 and 3 (Dvl1/2/3). Lipoproteins (TLR2 ligand), LPS (TLR4 ligand), flagellin (TLR5 ligand), bacterial CpG DNA (TLR9 ligand) and viral double-stranded RNA (TLR3 ligand).

The toll-like receptor (TLR) family consists of more than 13 members. All of them detect distinct pathogen-associated molecular patterns (PAMPs) derived from various microbial pathogens, such as viruses, bacteria, protozoa and fungi. The interaction of PAMPs with TLR culminates in the activation of nuclear factor kappa-light-chain-enhancer of activated B cells (NF-κB) through the Toll/IL-1 receptor (TIR)-domain-containing adaptors, myeloid differentiation primary response gene 88 (MyD88)- or TIR-domain-containing adapter-inducing interferon-β (TRIF)-dependent pathway, which controls the expression of inflammatory cytokine genes (9). The activated state of GSK3β promotes the activation of NF-κB, leading to a proinflammatory response; in contrast, activation of TLR2, 4, 5, 9 by the MyD88-dependent signaling pathway promotes the Akt (PKB)-dependent inactivation of GSK3β that leads to an anti-inflammatory response by inactivating NF-κB and activating the cAMP-response element binding protein (CREB), the activator protein 1 (AP-1), the signal-transducer and activator of transcription 1-3 (STAT1-3), the nuclear factor erythroid 2-related factor 2 (Nrf2), and β-catenin (6, 7, 10–13). During viral infection, activation of GSK3β by TLR3/TRIF signaling pathway controls the tumor necrosis factor (TNF) receptor associated factor 6 (TRAF6)-mitogen-activated protein kinase mitogen activated protein kinase kinase kinase (MAP3K7)-(TAK1) and receptor-interacting serine/threonine-protein 1(RIP1)/NF-κB axis to positively regulate pro-inflammatory cytokine production. Activation of GSK3β also activates the TRAF3-TRAF family member associated NFκB activator (TANK)-binding kinase 1 (TBK1)-interferon regulatory factor 3 (IRF3) axis to regulate IFN-β production (7). However, overexpression of constitutive active GSK3β results in the inhibition of NF-κB (14, 15) **(** **Figure 1** **)**.

Activation of the canonical Wnt signaling pathway has previously been shown to recruit Dishevelled (Dvl/Dsh) protein and enhances activation of GSK3β, thus influencing β-catenin degradation and toll-like receptor (TLR)/NF-κB signaling activation (16, 17). Also, Wnt5a/receptor tyrosine kinase-like orphan receptor 1 (ROR1)/Dvl2/Akt signaling activates NF-κB promoting the secretion of the cytokines, Interleukin (IL) 6 (IL-6), IL-11, and IL-18 (18). However, the regulatory effects of Wnt/β-catenin signaling are controversial because they depend on different contexts. For example, Wnt3a-frizzeled protein 1(Fzd1) interaction induces β-catenin accumulation and suppresses TLR/NFκB-dependent pro-inflammatory cytokine production (17). Recently, it was demonstrated in primary monocytes stimulated with lipopolysaccharide (LPS) and in a mice endotoxin model that Wnt3a/Dvl3 signaling functions as a negative regulator of TLR4-mediated inflammation through an increase of GSK3β phosphorylation at Ser9, the accumulation of β-catenin, and a subsequent suppression of NF-κB activity (10) **(** **Figure 1** **)**.

TNF receptor (TNFR)-associated intracellular signaling has been established as a pivotal activator of NF-κB and mitogen-activated protein kinases (MAPK) pathways. In addition, TNF signaling serves as negative regulator of noncanonical NF-κB and proinflammatory toll-like receptor (TLR) pathways (19). TNF-induced signaling promotes nuclear accumulation of GSK3β, which promotes anti-inflammatory response by mediating sustained expression of the signaling inhibitor A20 and IκBα synthesis that rapidly terminates TLR4-induced canonical NF-κB signaling, and by suppressing chromatin remodeling (20) **(** **Figure 1** **)**. Therefore, activation of TLRs, Frizzleds, and TNFR may lead to the active and inactive state of NF-κB, in the same way the active state of GSK3β leads to the active and inactive state of NF-κB **(** **Figure 1** **)**. All these different and complex responses make it hard to predict the end response of this transcriptional factor in the presence of an inflammatory stimulus. This is an open question and an exciting area for future research.

In order to update and discuss the most recent data published on modulation of the inflammatory response by GSK3β we searched for articles from 2012-2021 in the PubMed database that contained one of the following combination of keywords: GSK3beta inflammation bacterial infections (12 articles), GSK3beta inflammation lipopolysaccharide (104 articles), GSK3beta inflammation peptidoglycan (PGN) (6 articles), GSK3beta inflammation virus infection (25 articles), GSK3beta inflammation parasites infection (6 articles). In case of articles describing the role of GSK3β on inflammation caused by viruses and parasites we decided to include all articles published before and after 2012. Our first selection criteria were mainly based on articles containing original data obtained from cells or whole animal models infected with pathogenic bacteria, viruses and parasites or stimulated with purified PAMPs. Among these articles we only considered those with measurements of inflammatory molecules [e.g. IL-12 subunit p40 (IL-12p40), IL-1α/β, IL-6, tumor necrosis factor-α (TNF-α), nitric oxide (NO)] and, in some cases, those containing measurements of the anti-inflammatory molecules, such as IL-10. We have also included articles in which inflammation was reduced by modulation of GSK3β activity during infection with bacteria, viruses and parasites or PAMPs stimulation. It is worth to mention that this updated review does not include articles already cited in our previous article on the topic (21).

### GSK3β in the Inflammatory Response Activated by Bacteria Infection and LPS

In an animal model of keratitis it was observed that infection of corneal cells with *Pseudomonas aeruginosa* promoted GSK3β activation by decreasing its phosphorylation at Ser9, while the inhibition of GSK3β with SB216763, before *P. aeruginosa* infection, reduced the cornea inflammation by reducing the expression of IL-6 and IL-1β and by reducing the bacterial load (22). The traditional method for identifying *Escherichia coli* strains uses antibodies to test for surface antigens: The O-polysaccharide antigens, a component of LPS, flagellar H-antigens, and capsular K-antigens. There are currently ∼186 different *E. coli* O-groups and 53 H-types (23). Infection of HAEC HA549 with *Mycobacerium bovis* Bacillus Calmette-Guerin (BCG), a Gram-positive bacillus, or stimulation with LPS from unspecified origin (UO) exhibited TLR2/6 signaling activation and Wnt/β-catenin activity inhibition due to an increase in Axin and GSK3β expression. This combined effect led to a potent inflammatory response characterized by a robust expression of NF-κB and over-regulation of IL-6, IL-1α, IL-2, IL-8, and TNF-α (24). In MMC BV2 cell line activated by LPS (UO), it was observed an important induction of nitric oxide synthase (iNOS) protein expression and NO synthesis, which were reduced by LiCl and SB216763 (25). Moreover, based on the rat septic myocardial injury model, it was found that LPS (UO) induces GSK3β phosphorylation at its active site (Y216) and upregulates FOXO3A level in primary cardiomyocytes. The FOXO3A expression was significantly reduced by GSK3β inhibitors. *In vivo*, GSK3β suppression consistently improved cardiac function and relieved heart injury induced by LPS. In addition, the increase in inflammatory cytokines IL6, IL1β and TNFα in LPS-induced model was also blocked by inhibition of GSK3β, which curbed both ERK and NF-κB pathways, and suppressed cardiomyocyte apoptosis *via* activating the AMP-activated protein kinase (AMPK) (26). Long term exposure of male mice C57BL/6 to *E. coli* O26:B6 LPS in the neonatal period showed memory impairment and increased levels of TNFα and IL-1β as well as increased expression of GSK3β and Tau proteins in the hippocampus and cortex (27) (**Table 1**).

**Table 1:** GSK3β activity during modulation of the inflammatory response induced by bacterial, parasitic, LPS, viral infection, and viral-PAMP stimuli.

Stimulus or Animal model	Type of cell	GSK3β Inhibited: +pSer9 Activated: -pSer9 +pY216	NF-κB	β-catenin	Pro or anti-inflammatory molecules expressed	Pro or anti-inflammatory molecules suppressed	Ref.
*Pseudomonas aerugiosa*	Animal model of keratitis	SB216763	–	–	–	IL-6, IL-1β	(22)
*Mycobacterium bovis* or LPS UO	HAEC A-549 (Human)	GSK3-β^↑^	↑	↓	IL-6, IL-1α, IL-2, IL-8, TNFα	**-**	(24)
LPS (UO)	MMC BV2 (Murine)	LiCl, SB216763	**-**	↑	**-**	iNOS and NO	(25)
LPS (UO)	Cardiomyocytes	+pY216	**-**	**-**	IL-6, TNFα	**-**	(26)
LPS from *E. coli* O26:B6	Mice	GSK3-β^↑^	**-**	**-**	TNFα, IL-1β	**-**	(27)
*Porphyromonas gingivalis-*LPS	Human SCAP	+pSer9	**-**	**-**	IL-1β, TNFα	**-**	(28)
LPS (UO)	MCF7 and MDA-MB-231 (Human)	+pSer9	↑	↑	NF-κB*	**-**	(29)
LPS from *E. coli* O111:B4	Mice macrophages	-pSer9	**-**	**-**	**-**	IL-6, IL-12, TNFα	(30)
Protein p30 from HTLV-I	THP-1 monocytes (Human)	+pSer9	**-**	**-**	IL-10	MCP-1, TNFα, IL-8	(31)
MHV	3xTg-AD mouse model	-pSer9	**-**	**-**	MHCII^+^, CD4^+^, CD8^+^ TNF-α, IL-1β, IL-6	**-**	(32)
SeV	Mouse ESC and EFC	GSK-3β or GSK3α knockout	**-**	↓	**-**	IFNβ	(33)
HBV	Humans	+pSer9	**-**	↑	ALT, AST	**-**	(34)
*Leishmania donovani*	RAW264.7 mice BMDM	+pSer9	↓	↑	IL-10	IL-1β	(35)

(UO) Unspecified origin; (GSK3-β^↑^) Protein expression increased; (Inhibited function: ↑ Activated function: ↓); (*) Only NF-κB activation reported; (underline) Inflammation promoting cells increased; (Gray files) GSK3β inhibition promoted inflammation.

In contrast, human stem cells from the apical papilla (SCAP) stimulated with LPS from *Porphyromonas gingivalis*, a Gram-negative coccobacillus, showed an increase in phosphorylated GSK3β at Ser9 (phospho-GSK3β-Ser9), a residue recognized to inhibit the catalytic activity of the enzyme when it is phosphorylated, that resulted in the expression of IL-1β and TNF-α (28). LPS (UO) triggered TLR4/PI3K/Akt signaling pathway that resulted in phospho-GSK3β-Ser9 in MCF7 and MDA-MB-231 breast cancer cell lines. In addition, LPS promoted NF-κB p65 and p50 subunits nuclear translocation, suggesting an increase in the pro-inflammatory response (29). Also, a study in mice macrophages demonstrated that intracellular osteopontin, an inflammatory cytokine, negatively regulated the inflammatory response caused by LPS from *E. coli* O111:B4 *via* inhibition of GSK3β phosphorylation at Ser9 and activation of 4EBP1phosphorylation at Thr37/46 (30) **(** **Table 1** **)**.

It is important to note that the source of LPS used in some studies previously described was not specified. Probably, the LPS used come from different *E. coli* serotypes having different lipid A- or O-antigens. Therefore, we speculate that opposing inflammatory response observed when GSK3β is inhibited by phosphorylation at Ser9 may depend on the type of cell stimulated and/or the origin and composition of LPS, specifically the type of lipid A and/or O-antigen components.

### GSK3β in the Inflammatory Response Activated by Viruses

The transcription factor that binds to the purine-rich (PU)-box (PU.1) is a member of the E-twenty-six (*ETS)* family expressed exclusively in B lymphocytes, macrophages, and all hematopoietic lineages, except T-cell lines and mature T-lymphocytes (36). Interaction of the regulatory protein p30 from HTLV-I with PU.1 inhibited its DNA binding and transcription activity in THP-1 monocytes. This gave rise to a down-regulation of TLR4 number, an increase in phospho-GSK3β-Ser9, and a reduction of the pro-inflammatory cytokines TNF-α and IL-8, and monocyte chemoattractant protein-1 (MCP-1). HTLV-I p30 also stimulated the release of IL-10, an anti-inflammatory cytokine, after THP-1 monocytes down-regulation of TLR4 (31) (**Table 1**).

Infection of triple-transgenic mouse brain model 3xTg-AD with MHV caused a decrease in phospho-GSK3β-Ser9 that correlated with a strong increase in GSK3β activity, an increase in the number of cells expressing the major histocompatibility complex II (MHCII^+^), the cluster of differentiation (CD) 4 (CD4^+^), and 8 (CD8^+^), and an increase in the pro-inflammatory cytokines TNF-α, IL-1β, and IL-6 (32). The same pro-inflammatory response was observed when mice 3xTg-AD were treated with LPS from *E. coli* O55:B5. Altogether, these data suggest that viral- or bacterial-mediated infections trigger central nervous system inflammation that in turn may play a comorbidity factor for Alzheimer’s disease.

In mouse ESC and EFC cell lines infected with SeV GSK3α and GSK3β activated the antiviral innate immune response by phosphorylation of the β-catenin phosphodegron motif, which subsequently regulates interferon regulatory factor 3 (IRF3)-DNA binding and interferon β (IFN-β) gene expression (33). As GSK3β is considered a negative regulator of β-catenin, this study sheds light to this apparent paradox by demonstrating a different role of β-catenin phosphorylation by GSK3β in retinoic acid-inducible gene 1-like receptor (RLR) signaling. Furthermore, it also confirms that the GSK3β isoform activity alone is not sufficient for the antiviral response (33) **(** **Table 1** **)**. Injection of GSK3β inhibitors in mice caused an *in vivo* increase in CD8^+^ cytotoxic T lymphocyte (CTL) function. It was also observed a clearance of MHV68 and LCMV clone 13 and blocking of T cell exhaustion (37). This indicates that beneficial effects of GSK3β inhibitors in viral infections could be due to the activation of the immune system cells.

In humans, the aspartate transaminase (AST) and alanine transaminase (ALT) are specific indicators of liver inflammation and disease severity in a number of chronic liver diseases such as alcoholic and non-alcoholic liver disease, autoimmune liver disease and hepatitis infection (38). Interestingly, it was concluded that HBV infection enhanced β-catenin expression by activating the Akt/GSK3β signaling, which led to a marked increase in phospho-GSK3β-Ser9. Moreover, serum β-catenin levels correlated with elevated levels of ALT and AST but not with viral load, supporting the notion that serum β-catenin may be a useful tool for assessing HBV-related liver diseases (34) **(** **Table 1** **)**. It is likely that during viral infection the inhibited or activated state of GSK3β may promote inflammation, a similar situation to those observed in bacterial infections.

### GSK3β in the Inflammatory Response Activated by Parasites

*Leishmania* spp. is a parasite injected in the human blood as promastigotes by an insect that are phagocytized by macrophages (39). Studies in RAW264.7 murine macrophages and bone marrow-derived monocytes (BMDM) from mice infected with *Leishmania donovani* have demonstrated that GSK3β is phosphorylated and inhibited by Akt. As a consequence, GSK3β is no longer able to phosphorylates β-catenin and regulates the activation of forkhead box protein O1 (FOXO-1), a pro-apoptotic transcriptional regulator limiting both proinflammatory response and macrophage apoptosis. Macrophages transfected with a constitutively active GSK3β mutant and infected with *L. donovani* showed a decrease in parasite survival, reduction of IL-10 expression, and stimulation of IL-12 production. Collectively, these findings revealed that the intra-macrophage survival and multiplication of *L. donovani* is the result of host cell apoptosis and immune response inhibition (35) (**Table 1**). A similar situation, in which the inflammatory response is reduced by the GSK3β inhibited state was found in a rat sepsis model induced by either intravenous *E. coli* LPS or LPS plus *Staphylococcus aureus* peptidoglycan (PGN) (40, 41), when macrophages were stimulated with TLR2, 4, 5, 9 microbial agonists (12) and in other cellular types stimulated with PAMP or infected with bacteria (21). According to these published reports there still remain many open questions and it is evident that more studies are necessary to get a deeper insight on the function of GSK3β in the inflammatory response during parasite infection.

## Strategies to Inhibit the Inflammatory Response by GSK3β Inhibition

The GSK3β inhibitors so far designed for the treatment of infectious and non-infectious diseases such as diabetes, cancer, and neurodegenerative disorders have not been fully successful mainly because GSK3β is embedded in multi-protein complexes, which makes the access of inhibitory compounds difficult. Another problem concerns the almost structurally identical active sites of GSK3α and GSK3β. The lack of selectivity of the organic compounds designed against both GSK3 isoforms have excluded a range of promising GSK3 inhibitors from their journey into clinical trial phases (42). Nevertheless, a broad spectrum of GSK3β inhibitors as well as other inhibitors have been reported to inhibit GSK3β and inflammation during infection with bacteria, viruses and parasites as well as LPS and PGN cell stimulation. These inhibitors are classified within the following categories.

### ATP-Competitive and ATP-Noncompetitive Inhibitors of GSK3β

Among many competitive inhibitors against GSK3 (42), SB216763 is a potent, selective, and ATP-competitive GSK3α/β inhibitor with an IC_50_ of 34.3 nM for both isoforms (43). In a murine model of periodontal bone loss infected with *Porphyromonas gingivalis* and an *in vitro* study in MOLC MC3T3-E1 treated with LPS from *P. gingivalis* the inhibition of GSK3β with SB216763 before infection or LPS stimulation, induced the inhibition of the pro inflammatory cytokines IL-12p40, TNF-α, IL-1β, IL-6, and IL-17 expression (44, 45) **(** **Table S1** **)**. Interestingly, in mice treated with LPS from *E. coli* the compound SB216763 attenuated the NF-κB-mediated expression of IL-6 but not TNF-α (46). Moreover, in a murine acute liver failure (ALF) model induced by D-Galactosamine (D-GalN)/LPS (UO) the inhibition of GSK3β by SB216763 resulted in downregulation of TNF-α, IL-1β, and IL-12p40 expression (47, 48) **(** **Table S1** **)**.

The small molecule BIO is a highly potent, selective, and reversible ATP-competitive inhibitor of GSK3α/β with IC_50_ values around 5 nM. BIO maintains self-renewal in human and mouse ESC, regulates cell mass proliferation, and keeps the undifferentiated state of pancreatic MSC (49). Inhibition of GSK3α/β activity with SB216763 or BIO caused a delay in IκBα degradation and diminished the expression of TNFα in LPS (UO) stimulated neutrophils and macrophages. *In vivo* inhibition of GSK3α/β with SB216763 or BIO induced a decrease in the severity of LPS-induced lung injury as assessed by development of pulmonary edema, production of TNFα and macrophage inflammatory protein 2 (MIP-2), and release of the alarmins high-mobility group protein 1 (HMGB1) and histone H3 in lungs (50). In the context of viral infection, human hepatic CSCs (Huh7, JFH-1-Huh7, Huh7.5, and MH14C) treated with BIO and infected with HCV suffered an impairment of IFN signaling *via* inhibition of signal transducer and activator of transcription 1 (STAT1) phosphorylation and degradation (51). Also, a BIO analog 6BIGOE inhibited LPS (UO)-induced release of the pro inflammatory cytokines IL-1β, IL-6, and TNF-α, chemokine IL-8, and prostaglandin (PG) in human primary monocytes while increasing β-catenin and IL-10 levels *via* intracellular inhibition of GSK3 (52) **(** **Table S1** **)**.

Compound CHIR99021 is another cell-permeable, ATP-competitive inhibitor of GSK3 with IC_50_ values of 10 nM and 6.7 nM for GSK3α and GSK3β, respectively (53). CHIR99021 or SB216763 strongly reduced gene expression and secretion of the pro inflammatory cytokines TNF-α, IL-1β and IL-6, the chemokines IL-8 and MCP-1, the intercellular adhesion molecule 1 (ICAM-1), and the vascular cell adhesion molecule 1 (VCAM-1) in adipose tissue and skeletal muscle from women with gestational diabetes stimulated with LPS from *E. coli* 026:B6 (54) **(** **Table S1** **)**. In rats, the non-ATP competitive inhibitor thiadiazolidinone 8 (TDZD-8) or insulin treatment similarly reduced the plasma level of IL-1β and the organ injury/dysfunction caused by LPS from *E. coli* (O127:B8) plus PGN from *S. aureus* administration (55). Others non-ATP competitive inhibitors of GSK3β such as benzothiazepinones derivatives 3j and 6j highly attenuate *in vivo* the LPS (*E. coli* O55:B5)-induced acute lung injury (ALI) and diminish inflammation response in mice by inhibiting the IL-1β and IL-6 expression (56). Data from this study indicate that 3j and 6j might be potential candidates for further development of inflammation pharmacotherapy in LPS-induced ALI **(** **Table S1** **)**.

### Inhibition of the Inflammatory Response by Molecules that Induce Inhibition of GSK3β Activity by Phosphorylation

#### Plant Bioactive Compounds

Crude methanolic extracts (CME) from *Gleichenia truncata* used in malarial and melioidosis infection models showed anti-malarial and anti-inflammatory effects that were mediated in part by increased GSK3β phosphorylation at Ser9 (57). Apigenin is one of the most widespread flavonoids in plants (58). Apigenin from *Matricaria chamomilla* suppressed LPS (UO)-induced TNF-α, IL-1β, and IL-6 production in BV2 microglia *via* activating GSK3β/Nrf2 signaling pathway and suppressing NFκB activation (59). Also, Gastrodin, a natural phenol from *Gastrodia elata* BI, mediated anti-inflammatory and anti-proliferation effects in LPS (*E coli* O111:B4)-stimulated BV-2 or in primary microglia by modulating the Wnt/GSK3β/β-catenin signaling pathway (60). Phytochemicals such as ISO from *Inula helenium*, trigonoreidon B from *Rigonostemon reidioides*, betulin from the bark of birch trees, and xanthohumol from *Humululus lupulus* were able to induce inhibition of the inflammatory response by phospho-inhibition of GSK3β in BV2 and Raw 264.7 cells, and mice lung, respectively, treated with LPS from *E. coli* O55:B5 or LPS (UO) (61, 62–65). The phytopigments curcumin and anthocyanins exhibited anti-inflammatory activity in mice infected with the protozoan parasite *Plasmodium berghei* and also decreased the inflammatory response induced by LPS (UO) stimulation through an increase in phospho-GSK3β-Ser9 (66, 67) **(** **Table S1** **)**.

#### Proteins

Administration of the bioactive protein EPO to mice treated with LPS (UO) inhibited GSK3β and NF-κB activity, caused the reduction of the inflammatory cytokine IL-1β, and enhanced the formation of NO, which in turn caused local vasodilation, inhibited adhesion of platelets and neutrophils, and regulated angiogenesis (68, 69). Inhibition of GSK3β by recombinant human vaspin in HPMEC stimulated with *E. coli LPS* 0111:B4 promoted the reduction of mRNA expression levels of TNF-α, IL-6, VCAM, and E-selectin. In addition, mice subjected to systemic administration of adenoviral vector expressing vaspin were protected against LPS-induced acute respiratory distress syndrome by alleviating the pulmonary inflammatory response and pulmonary endothelial barrier dysfunction, which was accompanied by activation of the Akt/GSK3β pathway, leading to the phospho-inactivation of GSK3β (70) **(** **Table S1** **)**.

On the other hand, absence of active GSK3β and reduction of inflammation was observed in the myocardium of Tg mice overexpressing heat Shock 70 kDa protein 12B protein (HSPA12B) that were treated with LPS from *E. coli* O111:B4 (71). By manipulating the triggering receptor expressed on myeloid cells 2 (TREM2) levels with a lentiviral-mediated strategy, it was demonstrated in microglia that TREM2-overexpression following LPS (UO) stimulation led to a markedly reduction in GSK3β activity and tau hyperphosphorylation *via* suppression of the inflammatory response (72). In a mouse model of LPS from *E. coli* O55:B55-induced neuroinflammation, both gene deletion and pharmacological inhibition of the calcium-activated potassium channel KCa3.1, which is active in the phenotypic switch that occurs during astrogliosis in Alzheimer’s disease and ischemic stroke decreased CNS glia inflammation, including reactive astrogliosis and microglial activation *via* the Akt/GSK3β signaling pathway (73). Mice with deletion of ZNRF1 (another inflammation model) in their hematopoietic cells displayed an increased resistance to endotoxic and polymicrobial septic shock due to attenuated inflammation *via* the Akt–GSK3β pathway (74). On the other hand, Syk protein deficiency in MDC resulted in the suppression of LPS (*E. coli* O111:B4)-induced TNFα and IL-6 but enhancement of IFNβ and IL-10 due to GSK3β inactivation (75) **(** **Table S1** **)**.

Interestingly, the rLrp of *Mycobacterium tuberculosis* inhibited proinflammatory cytokine production and downregulated APC function in mouse macrophages *via* a TLR2-mediated PI3K/Akt pathway activation-dependent mechanism (76). Also it was identified that the protein GRA18 from *Toxoplasma gondii*, once released in Raw 264.7 macrophages cytoplasm, induced β-catenin up-regulation and the expression of a specific set of genes that are commonly associated with an anti-inflammatory response mediated by the chemokine C-C motif (CC) ligand(L)-17 (CCL-17) and CCL-22 by interacting with GSK3β/PP2A-B56 (77) **(** **Table S1** **)**.

#### Lipids and Lipid Derivatives

The α-lipoic acid, the RVD1/2 resolvins, and the maresin1 (MaR1) inhibit upregulation of VCAM-1, ICAM-1, iNOS, TNF-α, IL-1β, and IL-8 in LPS (*E. coli* O111:B4)-treated mice and LPS (*E. coli* O111:B4)-stimulated-BV-2 microglial cells, and primary human monocytes through a PI3K/Akt-dependent mechanism (78–80). Moreover, neutrophil LPC suppressed activation of NF-κB, leading to a decrease in the secretion of pro-inflammatory cytokines TNFα and IL-6, and an increased secretion of anti-inflammatory cytokine IL-10 through GSK3β phosphorylation during *M. tuberculosis* infection in mouse macrophages (81) **(** **Table S1** **)**.

#### Compounds Designed to Selectively Inhibit Proteins Structurally Unrelated to GSK3β

Reduction of inflammatory markers has also been observed by compounds designed to selectively inhibit proteins structurally unrelated to GSK3β in cells stimulated with LPS from *E. coli* O111:B4, O55:B5, and LPS (UO). Among these types of compounds, it is worth mentioning the cell permeable small molecule AMBMP that is a recognized canonical Wnt/β-catenin activator, the γ-secretase and notch inhibitor DAPT, and the novel benzoxazole derivative K313 with immunosuppressive activity toward T cell proliferation. They all inhibit the activity of GSK3β by increasing the relative abundance of phospho-GSK3β-Ser9 (82–84). **(** **Table S1** **)**, suggesting that these molecules may be used as effective GSK3β inhibitors of LPS-induced inflammation.

There are several FDA-approved compounds that indirectly modulate the inflammatory response in cell cultures stimulated with LPS from *E. coli* (UO) or PGN from *S. aureus* by increasing the relative abundance of phospho-GSK3β-Ser9. Some of these compounds are: S632A3, a glutarimide antibiotic; fluoxetine, an anti-depressive drug that selectively inhibits the serotonin reuptake; propofol, an enhancer of anesthesia used in surgical trauma; ephedrine hydrochloride, a vasopressor used to treat anesthesia-induced hypotension, sympathectomy or hypotension conditions derived from overdose of antihypertensive drugs; lithium, an element used to treat bipolar disorder that also acts as a tumor suppressor, and dexmedetomidina, that has sedative and analgesic properties (85–92) **(** **Table S1** **)**. Interestingly, it was demonstrated that progressively weighted ladder climbing as a rodent model of resistance-exercise training (RT) ameliorated LPS(UO)-induced cognitive impairment, a forerunner to neuroinflammatory diseases. These improvements in cognitive function occurred in concert with RT-induced IGF-1R/Akt/GSK3β signaling (93).

## GSK3β-Gene Knockdowns by siRNAs and miRNAs

Small interfering RNA (siRNA) and microRNA (miRNA) are short duplex RNA molecules that exert gene silencing effects at the post-transcriptional level by targeting specific messenger RNAs (mRNAs). However, their mechanisms of action and clinical applications are distinct. The major difference between siRNAs and miRNAs is that the former is highly specific to only one mRNA target, whereas the latter have multiple targets. Clinical trials of siRNA and miRNA-based drugs are already underway (94).

GSK3β-siRNA knockdown diminished expression of TNF-α in LPS(UO)-stimulated RAW 264.7 macrophages (50), inhibited NF-κB activation, enhanced CREB activation in LPS(UO)-stimulated acute monocytic leukemia THP-1 cells (95), and diminished expression of IL-1β, IL-6, and TNF-α in WNV infected HGC U251 cells (96). Silencing of phosphatase and tensin homolog (PTEN) and GSK3β with miR-21 induced macrophage efferocytosis and modulated LPS(UO)-induced inflammatory response (97). Recently, it was also shown that GSK3β-gene knockdown with miR-199b caused attenuation of the inflammatory response in THP-1 monocytes treated with LPS (*E. coli* O26:B6) (98) **(** **Table S1** **)**. Furthermore, IL-12p40 expression was evaluated by siRNA-gene expression silencing of GSK3α and GSK3β in BVE-E6E7 endothelial cells stimulated with PGN from *S. aureus*. Interestingly, GSK3α-gene silencing resulted in a marked increase in IL-12p40 while GSK3β-gene silencing had an opposite effect (99) **(** **Table S1** **)**. These data indicate that regulation of the inflammatory response by GSK3α or GSK3β may depend on the spatio-temporal regulation of both isoforms and the predominance of molecular mechanisms controlling their activity in each type of cell.

## Strategies to Inhibit the Inflammatory Response by GSK3β Activation

The phytochemicals salidroside and sappanone A induced inhibition of inflammation in mice stimulated with LPS (UO) through overexpression of GSK3β and reduction of the relative abundance of phospho-GSK3β-Ser9, respectively (100, 101). Isoproterenol inhibition of resistin or its siRNA-gene silencing in PDLC stimulated with nicotine and LPS from *P. gingivalis* had anti-inflammatory effects associated with activation of GSK3β and inactivation of β-catenin (102). The lipid derivative Mar-1 relieved inflammation in PDLC stimulated with LPS from *P. gingivalis* by GSK-3β activation and β-catenin expression inhibition (103). Interestingly, Nrf2 knockdown in RAW264.7 along with LPS (UO) stimulation caused an increase in the protein level of the glucose transporter type 4 and reduction of Akt and GSK3β phosphorylation. Nrf2 knockdown also induced a high-level secretion of IL6 and IL10. These results demonstrate that Nrf2 regulates inflammation and glucose metabolism besides its classic function in redox regulation (104) (**Table 2**).

**Table 2:** Treatments that inhibit the inflammatory response by GSK3β activation during LPS-stimulation.

Treatment used before or after infection	Type of cell or animal model	Microorganism or PAMP	Type of GSK3β activation	NF-κB Inhibited ↓	Nuclear factor activated ↑	Pro or anti-inflammatory molecule increased	Pro or anti-inflammatory molecule suppressed	Ref.
Salidroside	Rats	LPS (UO)	GSK3-β^↑^	**-**	**-**	**-**	TNFα, IL-6, IL-1β	(100)
Sappanone A	Mouse BMM	LPS (*UO)	-pSer9	**-**	**-**	**-**	Osteoclasts	(101)
Isoprotenerol, resisitin siRNA	Human periodontal ligament cells	LPS from *P. gingivalis* nicotine	-pSer9	↓	β-catenin **↓**	**-**	PGE2, NO, COX-2, TNFα, IL-1β, IL-6, IL-12, MMP-1, MMP-2, MMP-9	(33)
Maresin-1 (MaR1)	Human periodontal ligament cells (HPLC)	LPS from *P. gingivalis*	GSK3-β^↑^	**-**	**-**	**-**	IL-6, IL-8, TNFα, IL-1β	(103)
Nrf2 knockdown	RAW264.7	LPS (UO)	-pSer9	**-**	**-**	**-**	IL-6, IL-10	(104)

(GSK3-β^↑^) Protein expression increased; (-Ser9) GSK3β activated; (–) Not determined or not affected; (Underline) Inflammation promoting cells increased.

## Conclusions

**Figure 2:**
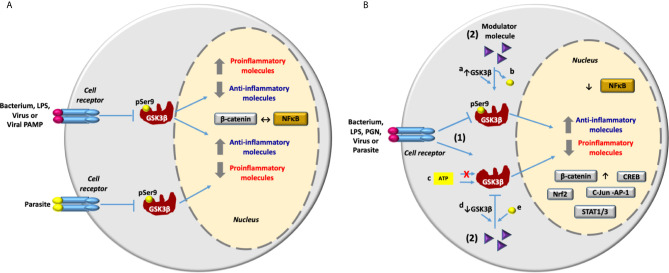
GSK3β in the inflammatory response activated by bacteria, viruses and parasites infection and lipopolysaccharide and viral PAMP stimulation. **(A)** An infectious stimulus can induce the inhibition of GSK3β through phosphorylation at Ser9. Consequently, depending mainly on the infectious stimulus, the expression of proinflammatory molecules can be inhibited or increased. The inflammatory response is carried out through the activation of the transcriptional factor NF-κB and the anti-inflammatory response is carried out through the activation of β-catenin. So that the activation of NF-κB inhibits the activation of β-catenin and vice versa (↔). **(B)** Inhibition of inflammation induced by infectious stimulus through modulation of GSK3β activity with different molecules. 1) An infectious stimulus induces inflammation through the inhibition or activation of GSK3β. 2) Molecules with different structural nature are able to control the inflammation modulating de activity of GSK3β by different mechanisms: By induction of a) the increase in GSK3β expression or b) dephosphorylation at Ser 9 [GSK3β activation] and by c) ATP-competitive and ATP-noncompetitive inhibition or by induction of d) the reduction in GSK3β expression, or e) phosphorylation at Ser9 [GSK3β inhibition]. Reduction in the expression of proinflammatory molecules and the increase in the anti-inflammatory molecules expression are carried out by inhibition of the transcriptional factor NF-κB and the activation of transcriptional factors β-catenin, Nrf2, CREB, cJun-AP1 and STAT1/3.

Data included in **Table 1** show that LPS from *P. gingivalis* and other type of LPS from different origin promoted opposing inflammatory responses on different cell types (HAEC A-549, MMC BV2, MCF7, MDA-MB-231, and Human SCAP) when GSK3β activity was inhibited. This variability may depend on the cell stimulated and/or the origin and composition of LPS used in these studies, specifically the lipid A and O-antigen components. The same phenotype was observed with viral stimuli. Human or murine cells stimulated with the viral PAMP, p30 protein or infected with viruses MHV or SeV, clearly shows that inhibition of GSK3β suppressed the inflammation. In contrast, data from a clinical study in humans infected with HBV indicate that inhibition of GSK3β also promoted inflammation **(** **Table 1** and **Figure 2** **)**. In regard to parasites, inhibition of GSK3β suppressed the inflammatory response in murine cells infected with *Leishmania donovani* **(** **Table 1** and **Figure 1** **)**. Further studies with different parasites and cells will allow to draw solid conclusions on the role of GSK3β in the inflammatory response.

Some of the studies included in **Table S1** and **Table 2** indicate that stimulation of human adipose tissue and skeletal muscle, RAW 264.7, and BV2 with LPS from *E. coli* serotype O55:B5 or stimulation of HPEC, mouse macrophages, mice BMDM RAW264.7, MDC, BV2, and primary human monocytes with LPS from *E. coli* serotype O111:B4 elicited a pro inflammatory response, which was suppressed through the inhibition of GSK3β with different molecules. In contrast to LPS from *E. coli* serotype 055:B5 and 0111:B4, LPS from *P. gingivalis* induced a proinflammatory response in human SCAP after inhibition of GSK3β **(** **Table 1** **).** Accordingly, LPS from *P. gingivalis* elicited a proinflammatory response in HPLC, which was suppressed through the activation of GSK3β with MaR1, isoprotenerol or resistin-siRNA **(** **Table 2** **)**. These apparently contradictory results indicate that inhibition of GSK3β may lead to inhibition or activation of the inflammatory response as it is the case when cells are stimulated with LPS from different sources **(** **Figure 2** **)**. Molecules with different structural nature were able to control inflammation through GSK3β inhibition **(** **Table S1** **).** However, we also observed that inflammation was inhibited by GSK3β overexpression (i.e. salidroside and maresin-1) or by decreasing the relative abundance of phospho-GSK3β-Ser9 (i.e. sappanone A, isoprotenerol or resistin-siRNA) **(** **Table 2** **)**. GSK3β signaling promoted by infection, activated NF-κB-mediated expression of proinflammatory molecules and inhibited the activity of β-catenin, Nrf2, CREB, STAT1/3, and cJun-AP1, except in MCF7 and MDA-MB-231 stimulated with LPS **(** **Tables 1**, **S1**, **2** and **Figure 2**). A related situation was found in human periodontal ligament cells stimulated with LPS from *P. gingivalis* and nicotine, and treated with isoprotenerol or resistin-siRNA. In this work, activation of GSK3β suppressed the NF-κB-dependent expression of proinflammatory molecules PGE2, NO, COX-2, TNF-α, IL-1β, IL-6, IL-12, MMP-1, MMP-2, MMP-9 and inhibited β-catenin activity **Table 2**.

Finally, data discussed in this review indicate that inhibition of GSK3β can induce a proinflammatory or anti-inflammatory response during infection, depending mainly on the microbial stimulus. Also, reduction of the inflammatory response does not always lead to GSK3β inhibition. Consequently, GSK3β should be considered as a switch to modulate inflammation. This is important when choosing the type of anti-inflammatory molecule required in each particular case and provides the basis to design new inhibitors.

## Author Contributions 

RC-V and VB-A conceived the idea and wrote the manuscript. OS-G, AG-G, SG-C, and C Á-A carefully and critically reviewed the manuscript. All authors contributed to the article and approved the submitted version.

## Funding

This work was supported by Coordinación de la Investigación Científica, Universidad Michoacana de San Nicolás de Hidalgo, Programa de Investigación 2020-2021.

## Conflict of Interest

The authors declare that the research was conducted in the absence of any commercial or financial relationships that could be construed as a potential conflict of interest.

## Glossary

**Table:**

Cytokines and chemokines	
IFN1 α/β	Interferon α/β
IL-1 α/β	Interleukin-1 subunit α and β
IL-2	Interleukin-2
IL-6	Interleukin-6
IL-8	Interleukin-8
IL-10	Interleukin-10
IL-12p40	Interleukin-12 subunit p40
IL-17	Interleukin-17
TNF-α	Tumor Necrosis Factor-α
Cell types	
BMDM	Bone marrow-derived macrophages
BVE-E6E7	Immortalized bovine endothelial cell line
CSC	Cancer Stem Cells
EFC	Embryonic fibroblast cells
ESC	Embryonic stem cells
ETS	Erythroblast transformation specific
HAEC A459	Human alveolar epithelial cells A459
HGC U251	Human glial cell line
HPMEC	Human pulmonary microvascular endothelial cells
PDLC	Human periodontal ligament cells
MCF7	Human mammary gland breast cells
MDA-MB-231	Human gland breast cells
MSC	Mesenchymal stem cells
MDC BV2	Murine dendritic cells BV2
MMC	Murine microglial cells
MOLC	Murine osteoblastic-like cells
SCAP	Stem cells from the apical papilla
THP-1	Human monocytes cell line
Viruses	
HBV	Human hepatitis B virus
HCV	Human hepatitis C virus
HTLV-I	Human T cell leukemia virus type I
LCMV	Lymphocytic choriomeningitis virus
MHV68	Murine gammaherpesvirus 68
MHV	Mouse hepatitis virus
SeV	Murine respirovirus, formerly Sendai virus
SARS-CoV-2	Severe acute respiratory syndrome coronavirus 2
Molecules that modulate inflammation through GSK3β regulation	
AMBMP	[2-Amino-4-(3,4-(methylenedioxy)benzylamino)-6-(3-methoxyphenyl) pyrimidine]
Apigenin	4´,5,7-trihydroxyflavone
Betulin	Lup-20 (28)-ene-3β, 28-diol
BIO	6-bromoindirubin-3´-oxime
BTZs 3j and 6j	Benzothiazepinones derivates
6BIGOE	6-bromoindirubin-3&rsquo;-glycerol-oxime ether
CHIR99021	(6-(2-(4-(2,4-Dichlorophenyl)-5-(4-methyl-1H-imidazol-2-yl)-pyrimidin-2-ylamino)ethyl-amino)-nicotinonitrile)
DAPT	[N-[(3,5- Difluorophenyl) acetyl]-L-alanyl-2-phenylglycine-1,1-dimethylethyl ester]
Dexmedetomidine	EPO
Erythropoietin	Gastrodin from Gastrodia elata BI
Gleichenia truncata crude methanolic extracts	GRA18
Toxoplasma dense granule protein 18	HSPA12B
Heat shock protein family A (Hsp70) member 12 B	ISO
11-deoxy-18α-glycyrrhetinic acid	isoalantolactone
K313	[1H-indole-2,3-dione 3-(1,3-benzoxazol-2-y1hydrazone)]
KCa3.1	Calcium-activated potassium channel
LiCl	Lithium chloride
LPC	Lysophosphatidylcholine
MaR1	Macrophage-derived mediator of inflammation maresin 1
Nrf2 knockdown	Nuclear factor erythroid 2-related factor 2 knockdown
SiRNA	Small interfering RNA
miRNA	microRNA
rLrp	Recombinant leucine-responsive regulatory protein
RVD1/2	Resolvins D1 and D2
Salidroside	(2-(4-Hydroxyphenyl)ethyl β-D-glucopyranoside
p-Hydroxyphenethyl	glucopyranoside Rhodioloside
Rhodosin	Tyrosol a-(β-D-glucopyranoside)
Sappanone A	((3E)-3-[(3,4-dihydroxyphenyl)methylidene]-7-hydroxychromen-4-one)
SB216763	((3-(2,4-Dichlorophenyl)-4-(1-methyl-1H-indol-3-yl)-1H-pyrrole-2,5-dione)
Syk knockdown	Spleen Tyrosine Kinase knockdown
TREM2	Triggering receptor expressed on myeloid cells 2
Xanthohumol	((E)-1-[2,4-Dihydroxy-6-methoxy-3-(3-methylbut-2-enyl)phenyl]-3-(4-hydroxyphenyl)prop-2-en-1-one)
ZNRF1 deletion mutant	Zinc And Ring Finger 1 deletion mutant
