# Supplementary material for: Glycogen Synthase Kinase 3β Modulates the Inflammatory Response Activated by Bacteria, Viruses, and Parasites
Source: Front Immunol. 2021 May 4;12:675751. doi: 10.3389/fimmu.2021.675751 (PMC8129516; doi:10.3389/fimmu.2021.675751)
Supplement: Supplementary file 1 [file Table_1.docx]

**Table 1S.** Treatments that inhibit the inflammatory response by GSK3β inhibition during infection with bacteria, viruses and parasites and bacterial PAMP-stimulation.

| **Treatment used before or after infection** | **Type of cell** | **Microorganism** | **Type of GSK3β inhibition** | **NF-κB** | **Nuclear** | **Pro or anti-inflammatory molecule increased** | **Pro or anti-inflammatory molecule suppressed** | **Ref.** |
| --- | --- | --- | --- | --- | --- | --- | --- | --- |
|  | **or animal model** | **or** |  | **Inhibited** | **Factor activated** |  |  |  |
|  |  | **PAMP** |  | **↓** | **↑** |  |  |  |
| SB216763 | Murine model of Periodontal bone loss | *Porphyromons gingivalis* | ATP-competition | **-** | **-** | **-** | IL-12 p40, TNF, IL-1β, IL-6, IL-17 | (44) |
|  |  |  |  |  |  |  |  |  |
| SB216763 | MOLC, MC3T3-E1 | *Porphyromons gingivalis-*LPS | ATP-competition | ↓ | β-catenin | **-** | IL-6, TNFα, IL-1β | (45) |
| SB216763 | Mice | Re-purified LPS from *E. coli* | ATP-competition | **-** | **-** | **-** | IL-6 | (46) |
| SB216763 | Murine ALF model | D-GalN and LPS (UO) | ATP-competition | ↓ | **-** | **-** | TNFα | (47) |
| SB216763 | Murine ALF model | D-GalN and LPS (UO) | ATP-competition | **-** | **-** | **-** | TNFα, IL-1β, IL-12p40 | (48) |
| BIO, SB216763 or siRNA | Neutrophils, | LPS (UO) | ATP-competition | ↓ | **-** | **-** | TNFα, MIP-2 | (50) |
|  | Mice induced ALI |  |  |  |  |  |  |  |
| BIO | Huh7, JFH-1-Huh7, Huh7.5, MH14C cells | HCV | ATP-competition | **-** | **-** | **-** | IFN | (51) |
| 6BIGOE | Human primary monocytes | LPS (UO) | ATP-competition | **-** | β-catenin | IL-10 | TNFα,IL-1 β, IL-6, IL-8, COX-2, PGE_2_ | (52) |
| CHIR9902123 SB216763 | Adipose tissue skeletal muscle from women with gestational DM | LPS from *E. coli* O26:B6 | ATP-competition | **-** | **-** | **-** | TNFα, IL-1β, IL-6, IL-8, MCP-1, ICAM-1, VCAM-1 | (54) |
| TDZD-8 or insulin | Rat | *E. coli* LPS (O127:B8) plus *S. aureus* PGN | Non-ATP competition | **-** | **-** | **-** | IL-1β | (55) |
| BTZs 3j and 6j | Mice | LPS from E. coli O55:B5 | Non-ATP-competition | **-** | **-** | **-** | IL-1β, IL-6 | (56) |
| *Gleichenia truncate* crude methanolic extracts | Murine model of malarial infection | *Plasmodium berghei* and *Burkholderia pseudomallei* | +pSer9 | **-** | **-** | IL-10 | TNFα, IFNγ | (57) |
| Apigenin from *Matricaria chamomilla* | BV2 | LPS (UO) | +pSer9 | ↓ | Nrf2 | HO-1 | TNFα, IL-1β, | (59) |
|  |  |  |  |  |  |  | IL-6 |  |
| Gastrodin from *Gastrodia elata* BI | BV2, primary microglia | LPS from *E. coli* O111:B4 | +pSer9 | **-** | β−catenin | **-** | iNOS and TNF-α | (60) |
| 11-deoxy-18α-glycyrrhetinic acid | Mice, rats, RAW 264.7 | LPS from *E. coli* O55:B5/D-galactosamine | +pSer9 | **-** | **-** | IL-10 | NO, TNFα, IL-6, IL-1β | (61) |
| Betulin | RAW 264.7 | LPS (UO) | +pSer9 | - | Nrf2 | - | iNOS, COX-2 | (64) |
| Xanthohumol | Mice | LPS O55:B5 | +pSer9 | ↓ | Nrf2 |  | iNOS, COX-2, TNFα, IL-6, IL-1β | (65) |
| Isoalantolactone (ISO) | BV2 | LPS from *E. coli* O55:B5 | +pSer9 | ↓ | Nrf2 | IL-10 | TNFα, IL-1β, NO, PGE_2_ | (62) |
| Trigonoreidon | RAW264.7 | LPS from *E. coli* O55:B5 | +pSer9 | ↓ | **-** | **-** | NO, PGE_2_, COX-2, IL-1β, TNFα, IL-6, IL-12 | (63) |
| Curcumin | Mice | *Plasmodium berghei* | +pSer9 | **-** | **-** | IL-10, IL-4 | TNFα, IFNγ | (66) |
| Anthocyanins | Mice | LPS (UO) | +pSer9 | ↓ | **-** | **-** | TNFα, IL-1β | (67) |
|  |  |  |  |  |  |  |  |  |
| EPO | Mice | LPS (UO) | +pSer9 | ↓ | **-** | **-** | IL-1β | (68, 69) |
| Vaspin | HPEC, mice lungs | LPS from *E. coli* O111:B4 | +pSer9 | ↓ | - | - | TNFα, IL-6, VCAM, E-selectin | (70) |
|  |  |  |  |  |  |  |  |  |
| HSPA12B-overspresing | Mice myocardium | LPS (UO) | +pSer9 | - | - | - | VCAM-1/ICAM-1 | (71) |
|  |  |  |  |  |  |  |  |  |
| TREM2-overexpression | Human microglia | LPS (UO) | GSK3β activity ↓ | - | - | - | TNFα, IL-6 | (72) |
|  |  |  |  |  |  |  |  |  |
| KCa3.1^−/−^ | Neuroinflammation mouse model | LPS from *E. coli* O55:B55 | +pSer9 | ↓ | - | - | TNFα, IL-1β | (73) |
|  |  |  |  |  |  |  |  |  |
| Znrf1^ϫ^ overexpressing | Mice, BMDM, RAW264.7 | LPS from *E. coli* O111:B4 | +pSer9 | ↓ | CREB | IL-10 | TNF, IL-6, IL-1β, CCL5 | (74) |
|  |  |  |  |  |  |  |  |  |
| Syk deficiency | MDC | LPS from *E. coli* O111:B4 | +pSer9 | ↓ | STAT1/3 | IL-10, IFN-β | IL-6, TNFα | (75) |
|  |  |  |  |  |  |  |  |  |
| rLrp from *Mycobacterium turberculosis* | Mouse macrophages | LPS from *E. coli* O111:B4 | +pSer9 | *↓* | - | - | TNF, IL-6, IL-12 | (76) |
|  |  |  |  |  |  |  |  |  |
| GRA18 | RAW264.7 | LPS | GRA18-GSK3β interaction | **-** | β-catenin | CCL-17, CCL-22 | **-** | (77) |
|  |  | *Toxoplasma gondii* |  |  |  |  |  |  |
| α-Lipoic acid | Mice | LPS from *E. coli* O111:B4 | +pSer9 | ↓ | **-** | **-** | VCAM-1, ICAM-1, iNOS | (78) |
| Lipoic acid | BV-2 | LPS from *E. coli* O111:B4 | +pSer9 | ↓ | **-** | **-** | iNOS, Nitrite | (79) |
| RVD1, RVD2, MaR1 | Primary human monocytes | LPS from *E. coli* O111:B4 | +pSer9 | ↓ | - | IL-10 | TNF, IL-1β, IL-8, IL-12 p40 | (80) |
|  |  |  |  |  |  |  |  |  |
| Neutrophil | Mouse macrophages | *Mycobacterium tuberculosis* | +pSer9 | ↓ | **-** | IL-10 | TNFα | (81) |
| Lysophosphatidylcholine |  |  |  |  |  |  |  |  |
| AMBMP | Human monocytes | LPS from *E. coli* O111:B4 | +pSer9 | ↓ | β-catenin | - | TNF, IL-6, IL-12 p40 | (82) |
|  |  |  |  |  |  |  |  |  |
| DAPT | BV-2 | LPS (UO) | +pSer9 | ↓ | - | IL-10 | IL-1β, TNFα, IL-6, iNOS, COX_2_, MCP-1 | (83) |
| K313 | RAW264.7 | LPS from *E. coli* O55:B5 | +pSer9 | - | - | - | NO, IL-6, TNFα | (84) |
|  |  |  |  |  |  |  |  |  |
| Fluoxetine | RAW264.7 | LPS from *E. coli* | +pSer9 | **-** | **-** | **-** | iNOS, NO, PGE2, COX-2 | (85) |
|  |  |  |  |  |  |  |  |  |
| Propofol | BV2 | LPS (UO) | +pSer9 | **-** | **-** | **-** | TNFα, IL-1β | (86) |
|  |  |  |  |  |  |  | IL-10 |  |
| *S632A3* | RAW264.7 | LPS (UO) | +pSer9 | ↓ | CREB | IL-10 | NO, PGE2, TNFα, IL-6 | (87) |
| Ephedrine hydrochloride (EH) | RAW264.7 | PGN from *Staphylococus auresu* | +pSer9 | - | - | IL-10 | IL-6, IL-12, IL-1β, TNFα | (88) |
|  |  |  |  |  |  |  |  |  |
| LiCl | Microglial cells | LPS (UO) | +pSer9 | - | β-catenin | IL-10, MRC1 | iNOS, IL-1β, IL-6, TNFα | (90) |
|  |  |  |  |  |  |  |  |  |
| Dexmedetomidine | Rats | LPS from *E. coli* 055: B5 | +pSer9 | ↓ | Nrf2, STAT3 | **-** | IL-1β, TNFα, IL-6, IL-18 | (91, 92) |
| siGSK3β | THP-1 cells | LPS (UO) | GSK3-β^↓^ | ↓ | **-** | **-** | **-** | (95) |
| siGSK3β | HGC U251 | *West Nile Virus* | GSK3-β^↓^ | ↓ | **-** | **-** | IL-1β, IL-6, TNFα | (96) |
|  |  |  |  |  |  |  |  |  |
| miRNA-21 | Post-efferocytotic macrophages | LPS (UO) | +pSer9 | ↓ | cJun-AP1 | IL-10 | TNFα | (97) |
|  |  |  |  |  |  |  |  |  |
| miR-199b | THP-1 | LPS from *E. coli* O26:B6 | GSK3-β^↓^ | ↓ | - | - | TNFα, IL-1β | (98) |
|  |  |  |  |  |  |  |  |  |
| siGSK3β | BVE-E6E7 | PGN from *S. aureus* | GSK3-β^↓^ | **-** | β-catenin | **-** | IL-12p40 | (99) |
|  |  |  |  |  |  |  |  |  |

(UO) Unspecified origin; (GSK3-β^↓^) Protein expression decreased; (+pSer9) GSK3β inhibited; (Inhibited function: ͎ ↑ Activated function: ↓); (-) Not determined or not affected.
